# Quantitative analysis of image quality for acceptance and commissioning of an MRI simulator with a semiautomatic method

**DOI:** 10.1002/acm2.12311

**Published:** 2018-03-24

**Authors:** Xinyuan Chen, Jianrong Dai

**Affiliations:** ^1^ Department of Radiation Oncology National Cancer Center/Cancer Hospital Chinese Academy of Medical Sciences and Peking Union Medical College Beijing China

**Keywords:** commissioning, MRI, simulation, simulator

## Abstract

Magnetic Resonance Imaging (MRI) simulation differs from diagnostic MRI in purpose, technical requirements, and implementation. We propose a semiautomatic method for image acceptance and commissioning for the scanner, the radiofrequency (RF) coils, and pulse sequences for an MRI simulator. The ACR MRI accreditation large phantom was used for image quality analysis with seven parameters. Standard ACR sequences with a split head coil were adopted to examine the scanner's basic performance. The performance of simulation RF coils were measured and compared using the standard sequence with different clinical diagnostic coils. We used simulation sequences with simulation coils to test the quality of image and advanced performance of the scanner. Codes and procedures were developed for semiautomatic image quality analysis. When using standard ACR sequences with a split head coil, image quality passed all ACR recommended criteria. The image intensity uniformity with a simulation RF coil decreased about 34% compared with the eight‐channel diagnostic head coil, while the other six image quality parameters were acceptable. Those two image quality parameters could be improved to more than 85% by built‐in intensity calibration methods. In the simulation sequences test, the contrast resolution was sensitive to the FOV and matrix settings. The geometric distortion of simulation sequences such as T1‐weighted and T2‐weighted images was well‐controlled in the isocenter and 10 cm off‐center within a range of ±1% (2 mm). We developed a semiautomatic image quality analysis method for quantitative evaluation of images and commissioning of an MRI simulator. The baseline performances of simulation RF coils and pulse sequences have been established for routine QA.

## 1 INTRODUCTION

Compared with CT, MRI has the advantages of nonionizing radiation, superior soft‐tissue contrast, and allowing quantitative or semiquantitative analysis of functional images.^1^, ^2^, ^3^ Developments in radiotherapy require precise MRI images for target and normal tissue delineation, characterizing tumor features, and monitoring treatment response during and after radiotherapy.^4^, ^5^ MRI simulation is a relatively new technique for radiotherapy.^6^, ^7^ Because MRI simulation serves a different purpose from MRI diagnosis, the technical requirements are different.^8^ To meet the needs of radiotherapy, an MRI simulator requires a scanning bore ≥70 cm, a flat couchtop, and an external laser positioning system installed in the scanner room.^9^

According to AAPM Report 100,^10^ the main procedures for acceptance and commissioning of a diagnostic MRI should include general system checks and MRI scanner system tests. Image quality tests play an important role in checking and monitoring the performances of an MRI scanner system. The gradient subsystem is assessed by geometric accuracy tests. Slice thickness accuracy is evaluated for combined gradient/radiofrequency (RF) subsystem. Percent image uniformity (PIU), high‐contrast spatial resolution (HCSR), low contrast detectability (LCD), and percent signal ghosting are evaluated for the performances of global system.

As of now, there is no formal technical report about the acceptance and commissioning of an MRI simulator. The image acceptance and commissioning for an MRI simulator are mostly described in AAPM Report 100,^10^ and the accuracies of laser and table are dealt with in AAPM Report TG 66.^11^ Several studies^12^, ^13^ have already reported and discussed the procedures and strategies of using an MRI simulator in radiation oncology department. However, overall strategies of image quality testing for acceptance and commissioning of MRI simulation still need to be explored.

The procedure of MRI simulation is complicated by comparison of CT simulation, because except the scanner, the RF coils also should be applied to receive the MR signal.^14^ The parameters setting for each pulse sequence in MRI scanning are also complicated and flexible.^15^ The image is dependent on a host of intrinsic parameter (the spin‐lattice relaxation time, the spin–spin relaxation time, etc.) and operator‐selectable parameters (repetition time (TR), echo time (TE), etc.). The image quality tests should not only reflect the hardware performance of the scanner but also reflect the features of the RF coils and pulse sequence. This may entail large quantities of image data for analysis at the MR workstation using built‐in measurement tools. The whole process involved quite a number of manual operation which will be time‐consuming and not easy to keep results objective enough.

MRI simulation differs from diagnostic MRI in purpose, technical requirements, and implementation. We are proposing a semiautomatic image acceptance testing and commissioning procedure for the scanner, simulation RF coils, and simulation pulse sequences of an MRI simulator.

## 2 MATERIALS AND METHODS

### 2.A An MRI simulator and testing phantom

Images were acquired on a 3.0 T 70 cm bore MRI scanner (GE Discovery MR750W, GE Healthcare, Wauwatosa, WI, USA). The maximum FOV is 50 cm. For our MRI simulator, an MRI‐compatible laser control system (DORADOnova MR3T LAP GmbH Laser Applikationen, Luneburg, Germany) was installed for positioning and simulation. A flat couchtop that supports three‐pin lock‐bars and MRI‐compatible positioning devices (GE Healthcare) were installed.

**Figure 1:**
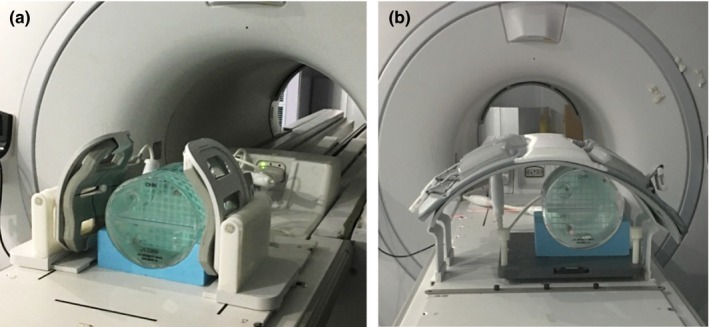
ACR Phantom setup. (a) Six‐channel simulation head coil; (b) 10 cm off‐center setup with simulation body arrays.

An ACR MRI accreditation phantom was used for image analysis. It is a cylindrical phantom with inside length 148 mm and inside diameter 190 mm. We designed a bracket for the phantom (Fig. 1) to stabilize its position on the flat couchtop.

According to Task Group No. 66 report, the accuracies of localization laser, external laser, table movement and couchtop should be tested before image commissioning.^11^ The localization lasers and external lasers were aligned with the center of the image plane using a laser alignment phantom (AQUARIUS Phantom, LAP Laser, Boynton Beach, FL, USA).

### 2.B Using ACR standard sequences with a split head coil

The MRI scanner includes static magnetic field subsystem, RF subsystem, and gradient subsystem. Acceptance testing and commissioning for the quality of the images generated by the scanner is conducted using a standard protocol using a split head coil and standard sequences prescribed by the ACR.^16^ It includes two axial spin echo standard acquisitions, a T1‐weighted image (T1WI) (TE/TR:20/500 ms) and a T2‐weighted image (T2WI) (TE/TR:80/2000 ms), with the settings FOV = 25 cm, thickness = 5 cm, gap = 5 cm, NEX = 1, matrix = 256 × 256. The scan direction is from chin to nose as labeled on the phantom, and the 11 standard scan layers for analyzing are labeled S1–S11.

### 2.C Testing simulation RF coils for radiotherapy with ACR standard sequences

Simulation RF coils for radiotherapy mainly include a special head coil and body arrays. The simulation head coil is a six‐channel phase‐array flex coil with separation designed for MRI simulation, which allows alignment of positioning devices [Fig. 1(a)]. For better comparison, diagnostic head coil with eight‐channel was also used in this study. The simulation body arrays are combined with the anterior and posterior arrays which are same as diagnosing body arrays in GE MR 750w platform. To be compatible with positioning devices and patients’ comfort, the posterior array is integrated into the couch and the anterior arrays are used with stands. The characteristics of images generated with different RF coils were tested with standard ACR T1WI and T2WI sequences.

### 2.D Testing clinical pulse sequences with simulation RF coils for radiotherapy

The commonly used simulation pulse sequences for radiotherapy are T1WI and T2WI, which were included in the study. The axial scanning with no slice direction interpolation was applied for all the sequences. With the simulation head coil, T1 fast spin echo (FSE), T1 three‐dimensional (3D) fast‐spoiled gradient recalled imaging (FSPGR), T2 Periodically Rotated Overlapping Parallel Lines with Enhanced Reconstruction (PROPELLER), and T2 FSE were tested using the ACR phantom. The slice thickness is often set at 2–3 mm for simulation of head and neck cancer or brain cancer cases. For better slice localization in the ACR phantom, the setting of 2.5 mm slice thickness with no spacing was adopted. FOV was set 25 cm, and frequency‐encoding direction was set at anterior/posterior (A/P). The other scanning parameters of simulation head sequences for commissioning are shown in Table 1.

**Table 1:** The main scanning parameters for simulation head sequences for the commissioning protocol

Sequences	TR (ms)	TE (ms)	Slices	ETL	RBW (KHz)	NEX	Matrix	Accel.(P/S)
T1 FSE	654	8.1	42	3	±41.67	2	320*256	2/1
T1 FSPGR	7.9	2.3	48	/	±31.25	1	320*320	2/1
T2 FSE	6006	108.2	42	16	±41.67	2	320*256	2/1
T2 PROPELLER	10165	87.4	42	28	±62.5	2	320*320	2/1

TR, repetition time; TE, echo time; ETL, echo train length; BW, bandwidth; NEX, number of excitation; Accel.(P/S), Acceleration (Phase/Slice).

For simulation body sequences, T1 in‐phase images of LAVA‐Flex (Liver Acquisition with Volume Acceleration with Flex processing), T1 FSE, T2 PROPELLER, T2 FSE were included in the study with FOV = 42 cm, slice thickness = 5 mm, and gap = 0, and frequency‐encoding direction was set at right and left (R/L). The other scanning parameters are shown in Table 2. For testing the whole FOV for geometric accuracy, the ACR phantom was installed in both the isocenter and 10 cm off‐center.

**Table 2:** The main scanning parameters of simulation body sequences for the commissioning protocol

Sequences	TR (ms)	TE (ms)	Slice	ETL	RBW (kHz)	NEX	Matrix	Accel. (P/S)
T1 FSE	470	6.8	21	3	±62.5	2	352*352	2/1
T1 LAVA‐Flex	4.6	2.2	28	/	±142.86	2	256*256	2/1.25
T2 FSE	3000	105.8	21	16	±62.5	2	352*352	2/1
T2 PROPELLER	9398	110.7	21	28	±62.5	2	352*352	2/1

### 2.E Image analysis

Seven quantitative image parameters were tested and recorded for MR simulation image acceptance and commissioning.

For geometric accuracy, the diameters of four radial lines (0°, 90°, ±45°) on S5 and S1 were auto‐measured. The percent geometric distortion (%GD) was calculated separately according to the following equation:

(1) $$\%GD=\frac{actual\;\;di\;mension-measured\;dimension}{actual\;dimension}\times 100$$

The slice position accuracy tested on S1 and S11 was auto‐calculated with the difference of left and right bars separately (Eq. (2)). Half of ΔSP was the actual slice displacement error. When ΔSP >0, the slice mispositions superiorly, and ΔSP <0 means the slice mispositions inferiorly.

(2) ΔSP=leftbar−rightbar

Slice thickness (ST) in MRI is ideally determined by the bandwidth of the RF excitation pulse and the amplitude of the associated applied gradient pulse. A pair of 10:1 crossed signal ramps with negative and positive slope was used to measure ST. The ST was calculated automatically with average length of top and bottom signal ramps on S1 (Eq. (3)).

(3) $$ST=0.2*\frac{top*bottom}{top+bottom}$$

The percent integral uniformity (PIU) for image intensity was calculated according to Eq. (3), below. A 1 cm^2^ circular region of mean maximum $\left({\overset{¯}{S}}_{\max}\right)$ and minimum $\left({\overset{¯}{S}}_{\min}\right)$ gray values within the center region of a 200 cm^2^ circle on S5 was automatically delineated and recorded.

(4) $$PIU=100∙\left[1-\frac{{\overset{¯}{S}}_{\max}-{\overset{¯}{S}}_{\min}}{{\overset{¯}{S}}_{\max}+{\overset{¯}{S}}_{\min}}\right]$$

For testing percent signal ghosting, four rectangular regions of 10 cm^2^ were delineated for extracting mean signals in the frequency‐encoding direction (${\overset{¯}{S}}_{FE1}$ and ${\overset{¯}{S}}_{FE2}$) and in the phase‐encoding direction (${\overset{¯}{S}}_{PE1}$ and ${\overset{¯}{S}}_{PE2}$). The mean signal ($\overset{¯}{S}$) of the 200 cm^2^ circle within the center region was also recorded. The ghosting ratio was calculated as:

(5) $$GR=\left|\frac{\left({\overset{¯}{S}}_{FE1}+{\overset{¯}{S}}_{FE2}\right)-\left({\overset{¯}{S}}_{PE1}+{\overset{¯}{S}}_{PE2}\right)}{2\overset{¯}{S}}\right|$$

HCSR in frequency‐encoding and phase‐encoding directions was semiauto tested on S1. An experienced medical physicist identified three pairs of arrays of holes with resolutions of 1.1, 1.0, and 0.9 mm, respectively.

LCD was semiauto tested from S8 to S11, which represented the contrast at 1.4%, 2.5%, 3.6%, and 5.1%, respectively. The value of LCD was the sum of the number of complete spokes on each slice.

**Figure 2:**
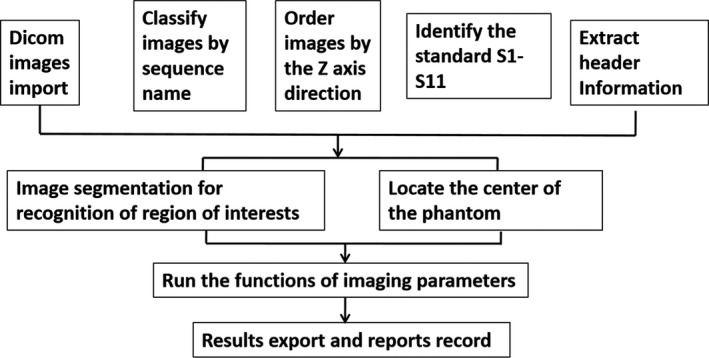
The whole procedure of semiautomatic analysis.

For ensuring consistency and reproducibility of the results, and improving the efficiency of analysis, in‐house codes and procedures were developed for semiautomatic image analysis (Fig. 2) using Matlab (R2014a, The Mathworks, Natick, MA, USA). The whole process included four modules, i.e., automatic Dicom data processing, automatic image recognition, semiautomatic parameters calculation, and automatic results record. The functions for automatically calculating the five quantitative parameters (including %GD, ΔSP, ST, PIU, and GR) were developed based on Eqs. (1), (2), (3), (4), (5), separately. Semiautomatic methods were also established to analyze HCSR and LCD. The accuracy of codes compared with manual analysis has been validated before clinical use.

## 3 RESULTS

### 3.A Using ACR standard sequences with the split head coil

**Figure 3:**
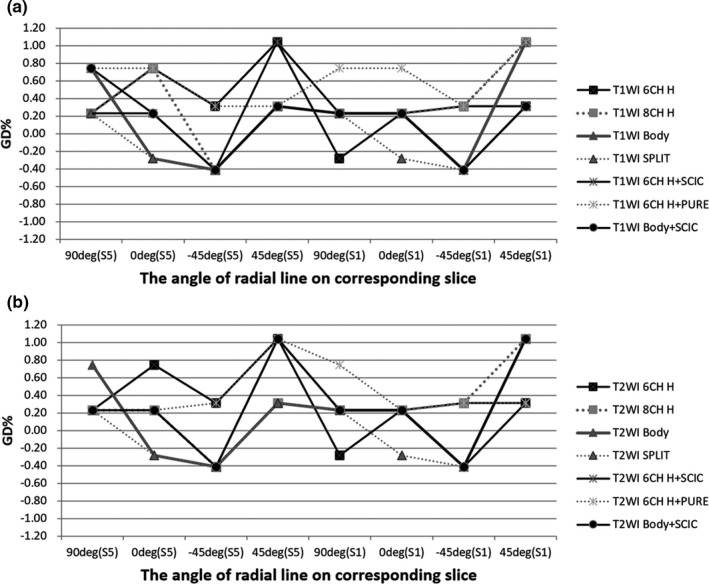
The % GD of radial lines at different angles using ACR standard sequences with different coils and setup: (a) T1WI; (b) T2WI.

**Figure 4:**
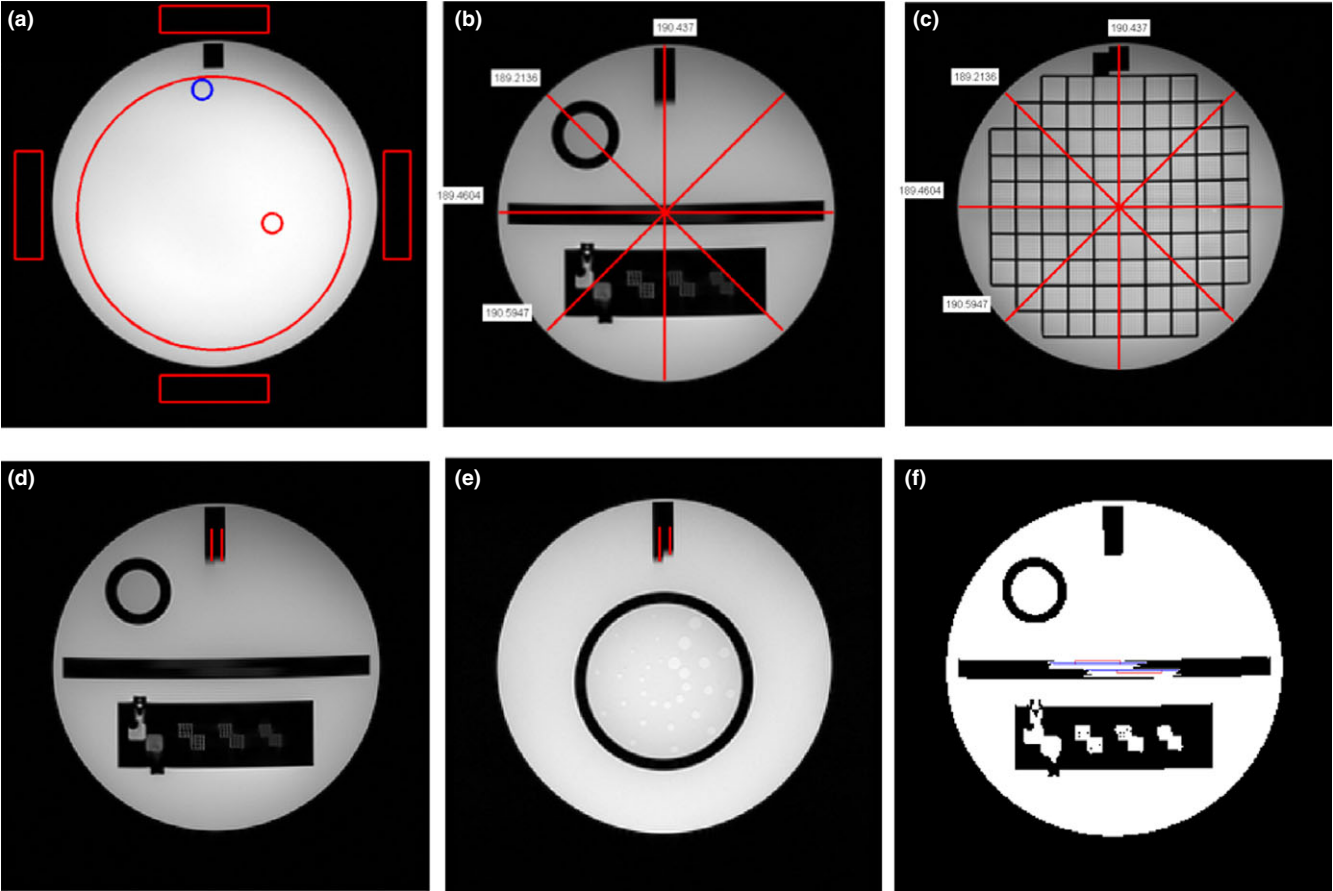
The automatically calculated image parameters for ACR T1WI with split head coil: (a) PIU and GR on S7; (b) and (c) Geometric accuracy on S1 and S5; (d) and (e) slice position accuracy on S1 and S11; (f) slice thickness accuracy on S1.

According to the AAPM 100 report, all the image parameters using ACR standard sequences with a split head coil pass the threshold of acceptance criteria (Table 3 and Fig. 3). Figure 4 shows the automatically calculated image parameters for ACR T1WI sequence.

**Table 3:** ACR standard sequences with the split head coil with acceptance criteria

Sequences	PIU%	GR%	ST	SP (S1/S11)	LCD	HCSR (LR/AP)	Time (min)
T1WI	88.76	0.16	5.08	0.00/‐3.91	40	1/1	2.27
T2WI	89.51	0.14	5.47	1.95/‐0.98	40	1/1	8.93
(Criteria)	≥82%	±2 mm	±10%	±5 mm	≥37	<1/1	

LR, left and right direction; AP, anterior and posterior direction.

### 3.B Testing simulation RF coils for radiotherapy with ACR standard sequences

The performance of the six‐channel simulation head coil, eight‐channel diagnostic head coil, and the simulation body arrays were tested by conducting axial ACR standard sequences.

**Figure 5:**
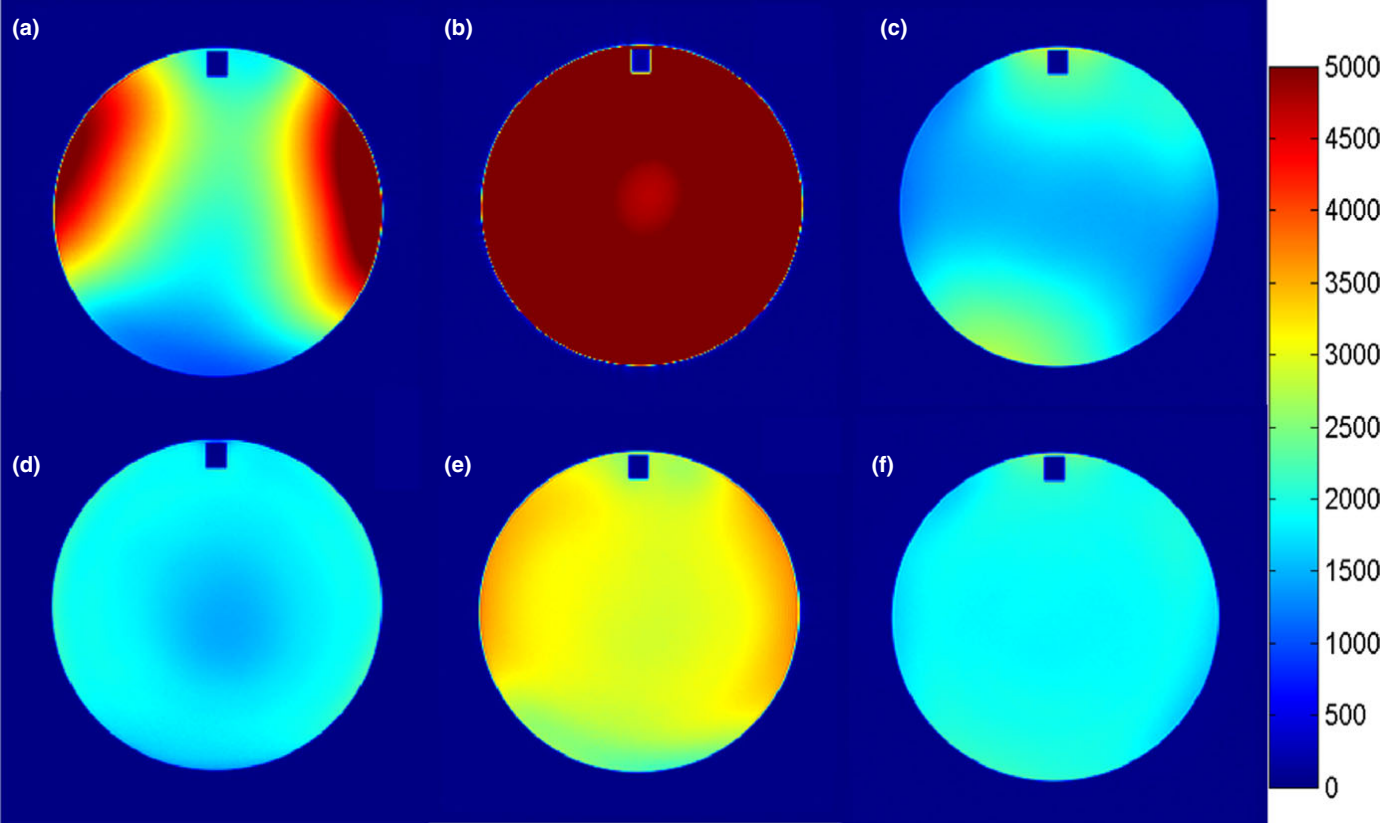
PIU testing: (a) Six‐channel simulation head coil; (b) eight‐channel diagnostic head coil; (c) simulation body arrays; (d) six‐channel simulation head coil + PURE; (e) six‐channel simulation head coil + SCIC; f) simulation body arrays + SCIC.

For ACR T1WI and T2WI sequences, compared with the eight‐channel diagnostic head coil, the PIU of the simulation head coil decreased by 34.37% and 34.04%, respectively (Table 4). To improve the image uniformity, the built‐in calibration method phase‐array uniformity enhancement (PURE) or surface coil intensity correction (SCIC) was chosen, and all the other scanning parameters were kept unchanged. For the six‐channel simulation head coil, image uniformity of ACR T1WI increased to 86% and 89.49%, and of ACR T2WI increased to 85.44% and 89.83%, respectively (Table 5 and Fig. 5).

**Table 4:** Testing different clinical RF coils with ACR standard sequences

Sequences	Coil	PIU%	GR	ST	SP (S1/S11)	LCD	HCSR (LR/AP)	Time (min)
T1WI	6CH H	39.17	0.21	5.28	−0.98/−3.91	38	1/1	2.27
	8CH H	73.53	0.01	5.40	1.95/−3.91	40	1/1	2.27
	Body	68.93	0.16	5.24	2.93/−2.93	40	1/1	2.27
T2WI	6CH H	38.97	0.00	5.31	−0.98/−3.91	38	1/1	8.93
	8CHH	73.01	0.28	5.37	1.95/−3.91	40	1/1	8.93
	Body	69.19	0.06	5.32	2.93/−2.93	40	1/1	8.93

6CH H, six‐channel simulation head coil; 8CH H, eight‐channel diagnostic head coil; Body, simulation body arrays.

**Table 5:** Image parameters using intensity calibration for six‐channel simulation head coil

Sequences	Intensity correction	PIU%	GR	ST	SP (S1/S11)	LCD	HCSR (LR/AP)	Time (min)
T1WI	PURE	86.00	0.10	5.27	0.00/−2.93	39	1/1	2.27
	SCIC	89.49	0.16	5.15	−0.98/−2.93	39	1/1	2.27
T2WI	PURE	89.75	0.89	4.85	−0.98/−2.93	40	1/1	8.93
	SCIC	85.44	0.09	5.26	0.98/−3.91	38	1/1	8.93

For ACR T1WI and T2WI sequences with the six‐channel simulation head coil, LCD was 37. Only seven spokes could be seen on S8 of the 1.4% contrast module. The reason is that the poor uniformity made three spokes difficult to fully visualize. When using the SCIC or PURE methods to calibrate the intensity, LCD values also could not fully reach 40. In the test of HCSR, the values of LR and AP were all 1 by using three kinds of coils. GR for all coils could be controlled <2.5%. On S1 and S5, the average %GD was controlled in the range of ± 1%. Slice position could be controlled within ±0.4 cm on Slice 1 and Slice 11 by using all coils. The ST for each coil also could be controlled in the normal range.

### 3.C Testing simulation pulse sequences with simulation RF coils for radiotherapy

**Figure 6:**
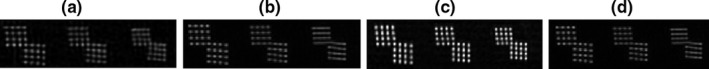
HCSR for six‐channel simulation head coil: (a) T2 propeller (0.9/0.9); (b) T2 FSE (0.9–1/0.9); (c) T1 3D FSPGR(0.9/0.9); (d) T1FSE(0.9–1/0.9).

**Figure 7:**
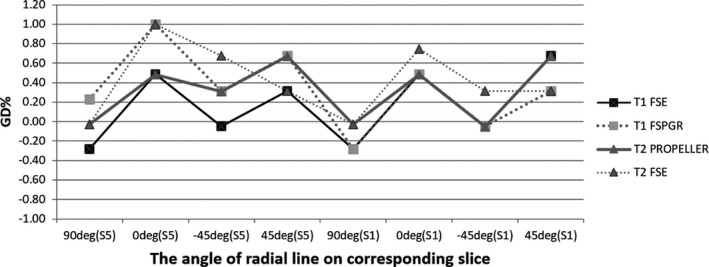
Geometric accuracy test for simulation pulse sequence with six‐channel simulation head coil.

Table 6 shows the summary of image quality parameters of T1WI and T2WI clinical pulse sequences by using the six‐channel simulation head coil. With regard to PIU, the values of all the sequence were <40%, and the mean value with standard deviation was 36.68 ± 0.57%, which was similar to the results of the ACR standard sequences using the same coil. For HCSR, the values in some of the clinical sequences are improved less than 1. The matrix in the phase‐encoding direction was set less than in the frequency‐encoding direction; the corresponding HCSR is 0.9–1 in the phase‐encoding direction and 0.9 in the frequency‐encoding direction (Fig. 6). For the LCD, the values of T2‐weighted simulation pulse sequences were lower than of T1‐weighted simulation pulse sequences. The slice thickness of 3D T1 FSPGR was 35.2% higher than the true value of 2.5 mm. The scanning time of 3D T1 FSPGR was the shortest among the T1WI sequences. The parameters of slice position, geometric accuracy, and GR were in the normal range (Table 6 and Fig. 7).

**Table 6:** Image parameters for testing clinical pulse sequence with six‐channel simulation head coil

Sequences	PIU	GR%	ST	SP (S1/S11)	LCD	HCSR (LR/AP)	Time (min)
T1 FSE	36.96	2.49	2.75	−0.49/−2.44	36	0.9–1/0.9	2.18
T1 FSPGR	36.92	0.51	3.38	−0.49/−1.95	36	0.9/0.9	1.02
T2 FSE	35.75	0.93	2.66	−0.49/−2.93	28	0.9–1/0.9	2.27
T2 Propeller	37.89	0.33	2.90	0.00/−2.44	27	0.9/0.9	2.25

**Figure 8:**
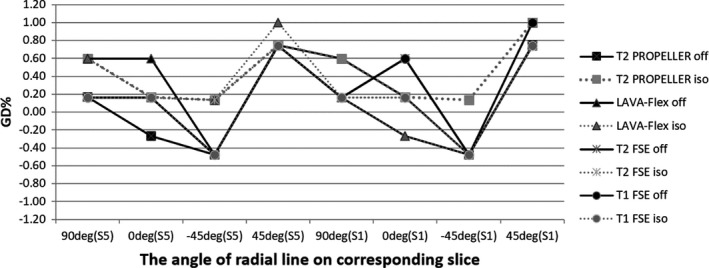
Geometric accuracy test for simulation pulse sequences with simulation body arrays in isocenter and 10 cm off‐center.

Table 7 shows the image quality results of clinical pulse sequences using body arrays. Due to the large FOV, the HCSR for all clinical sequences are <1, and for the LAVA‐flex sequence, the HCSR could not reach 1.1. The LCD in LAVA‐flex was only 20, and in T2 PROPELLER and T2 FSE were 33. Owing to the low, ramp signal, the slice thickness could not be measured accurately for LAVA‐flex. The slice thickness of other sequences could be controlled in the range of 5 mm ± 0.5 mm. The results of PIU were similar to the ACR standard sequences using the same coils, with a mean value of 67.73 ± 1.57%. The geometric accuracy for isocenter and off‐center was controlled within ±1% (Fig. 8). The slice position for all tested sequences also was kept in the normal range. The scan time of LAVA‐Flex is the shortest among the tested clinical sequences.

**Table 7:** The image quality results of simulation pulse sequences by using simulation body arrays

Sequences	PIU (%)	GR (%)	ST	SP (S1/S11)	LCD	HCR (LR/AP)	Time (min)
T1FSE	69.80	0.21	5.29	3.28/−3.28	35	1.1/1.1	2.07
LAVA‐Flex	67.19	0.22	NA	−3.28/3.28	20	>1.1/>1.1	0.52
T2FSE	68.81	0.39	4.58	1.64/−3.28	33	1.1/1.1	1.3
PROPELLER	65.13	0.20	4.97	2.46/−4.69	33	1.1/1.1	2.3

## 4 DISCUSSION

With the growing prevalence of MRI simulators in radiation oncology departments, it is imperative to monitor the stability of scanners, RF coils, and the clinical RF sequences. The image acceptance testing and commissioning should include these three parts, using the standard sequence with a split head coil to test the basic performance of scanner, examining the features of simulation RF coils with the standard sequences, and using simulation sequences with simulation RF coils to test the character of the sequence and advanced performance of the scanner. The AAPM‐associated tolerances are appropriate only for standard test protocol. To monitor the performances of simulation RF coils and pulse sequences, the baseline should be established by acceptance testing and commissioning for routine QA. Most of the image quality parameters can be tested using the ACR phantom to ensure consistency and reproducibility of the whole workflow. Before applying new simulation sequences for the purpose of radiotherapy, the character of images should be carefully tested.

The intensity uniformity of MRI system is due to both the RF transmitting (B1‐field) and RF receiving systems. The intensity uniformity of images using conventional diagnostic coil also could not meet the tolerance recommended by AAPM. That is because, the surface coil may lead to lose some image uniformity, although it is characterized by a high signal‐to‐noise ratio.^17^, ^18^ However, compared with the conventional diagnostic head coil, the six‐channel simulation head coil produces more serious heterogeneity. Conducting simulation sequences with the simulation head coil, the PIU is still quite low. Liney et al.^12^ reported that the radiation head coil was less homogeneous than the GE head and neck coil and the body phase array. The intensity correction method is recommended to be chosen when using six‐channel simulation head coil. The basic theory of SCIC correct method is image postprocessing (like smoothing and filtering). Applying PURE method for uniformity correction need calibration scan to generate correction map. It should be noted that correction methods still could not support all the pulse sequences right now.

**Figure 9:**
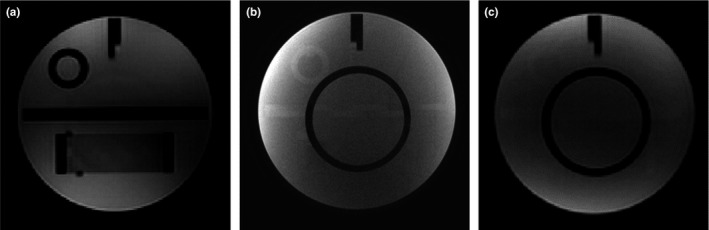
Wrap artifact of 3D scanning: (a) the first slice of LAVA‐flex; (b) the last slice of LAVA‐flex; (c) the last slice of 3D FSPGR.

The structure of the human body is obviously much more complicated than that of a phantom. Some features of sequences cannot be tested by phantom only. To determine the clinical pulse sequences for radiotherapy, the tests should be performed both in a phantom and in vivo. In our study, the commonly used clinical sequences were included in the phantom test. The high‐contrast resolution is sensitive to the setting of FOV and matrix. LAVA is a 3D spoiled gradient echo technique with extensive coverage and rapid acquisition, but compromises high‐ and low‐contrast resolution to some extent. Due to the wrap artifact in the pattern of 3D scans (FSPGR and LAVA‐Flex) as shown in Fig. 9, the number of slices should be increased. The slice thicknesses of the FSPGR and LAVA are not accurate enough compared with the FSE sequence. The image quality also depends upon parameters such as flip angle, ETL, TR, and other scan parameters that may affect the results of LCD in phantom tests.

Geometric distortion is complex and depends on many factors including system imperfection, patient anatomy, pulse sequence type, and image parameters.^19^ System‐dependent distortion mainly stems from gradient nonlinearities, static field inhomogeneities, and eddy currents created by the switching of field gradients and maladjustments of both the gradient offsets and the radio frequency.^20^ For the purpose of radiotherapy, it is particularly important to check whether the geometrical accuracy of image is sufficient to allow precise target and OARs delineation. The assessment of geometric distortion for MR simulation images should include the whole FOV. In this study, we placed the phantom in the isocenter and 10 cm off‐center to test the GD using simulation body arrays. And GDs of two positions can be all controlled in the range of ±1% (2 mm). Some new large geometric phantoms are being developed to simulate and evaluate distortion with large FOV.^21^, ^22^ The magnitude of the distortions increases with increasing distance from the isocenter of the scanner.^23^, ^24^ Similar to our results, within a distance of 200 mm, the mean distortion in the axial plane can be controlled in an acceptable range for radiotherapy. Distortion in the sagittal and coronal planes can also be evaluated using the ACR large phantom.

The newly developed semiautomatic image analysis codes could make the results of measurement consistent, reduce the bias of human analysis, and save time. They improve the precision and efficiency of the acceptance test, and could be a useful software tool for routine QA procedures. The automatic image analysis procedure for both MRI and cone beam can help finish the uniform acceptance and constancy testing.^25^, ^26^ Fully automatic image quality assurance for an MRI simulator system should be carefully considered in the future to minimize manual intervention.

## 5 CONCLUSION

Following the AAPM Report 100 for MRI, we developed a semiautomatic method to evaluate the basic image quality parameters for an MRI simulator. A series of image acceptance tests and commissioning for the scanner, RF coils, and pulse sequences have been constructed. The six‐channel simulation head coil can provide comparable images, except for poor uniformity. The intensity correction method is recommended to be chosen when using six‐channel simulation head coil. The baseline performances of simulation RF coils and pulse sequences have been established for routine QA. These proposed procedures can be added as the part of an MRI simulator commissioning.

## CONFLICT OF INTEREST

All authors approved the final manuscript, and declared that they have no potential conflicts of interest to this work.
